# Quantitative Evaluation of COVID-19 Pneumonia Lung Extension by Specific Software and Correlation with Patient Clinical Outcome

**DOI:** 10.3390/diagnostics11020265

**Published:** 2021-02-09

**Authors:** Andrea Daniele Annoni, Edoardo Conte, Maria Elisabetta Mancini, Carlo Gigante, Cecilia Agalbato, Alberto Formenti, Giuseppe Muscogiuri, Saima Mushtaq, Marco Guglielmo, Andrea Baggiano, Alice Bonomi, Mauro Pepi, Gianluca Pontone, Daniele Andreini

**Affiliations:** 1 Centro Cardiologico Monzino IRCCS, 20138 Milan, Italy; edoardo.conte@ccfm.it (E.C.); maria.mancini@ccfm.it (M.E.M.); gigante.carlo@gmail.com (C.G.); cecilia.agalbato@ccfm.it (C.A.); alberto.formenti@ccfm.it (A.F.); giuseppe.muscogiuri@ccfm.it (G.M.); saima.mushtaq@ccfm.it (S.M.); marco.guglielmo@ccfm.it (M.G.); andrea.baggiano@ccfm.it (A.B.); alice.bonomi@ccfm.it (A.B.); mauro.pepi@ccfm.it (M.P.); gianluca.pontone@ccfm.it (G.P.); daniele.andreini@ccfm.it (D.A.); 2 Department of Clinical Sciences and Community Health, University of Milan, 20122 Milan, Italy

**Keywords:** coronavirus infections, pneumonia, lung, computed tomography

## Abstract

Lung infection named as COVID-19 is an infectious disease caused by the most recently discovered coronavirus 2 (SARS-CoV-2). CT (computed tomography) has been shown to have good sensitivity in comparison with RT-PCR, particularly in early stages. However, CT findings appear to not always be related to a certain clinical severity. The aim of this study is to evaluate a correlation between the percentage of lung parenchyma volume involved with COVID-19 infection (compared to the total lung volume) at baseline diagnosis and correlated to the patient’s clinical course (need for ventilator assistance and or death). All patients with suspected COVID-19 lung disease referred to our imaging department for Chest CT from 24 February to 6 April 2020were included in the study. Specific CT features were assessed including the amount of high attenuation areas (HAA) related to lung infection. HAA, defined as the percentage of lung parenchyma above a predefined threshold of −650 (HAA%, HAA/total lung volume), was automatically calculated using a dedicated segmentation software. Lung volumes and CT findings were correlated with patient’s clinical course. Logistic regressions were performed to assess the predictive value of clinical, inflammatory and CT parameters for the defined outcome. In the overall population we found an average infected lung volume of 31.4 ± 26.3% while in the subgroup of patients who needed ventilator assistance and who died as well as the patients who died without receiving ventilator assistance the volume of infected lung was significantly higher 41.4 ± 28.5 and 72.7 ± 36.2 (*p* < 0.001). In logistic regression analysis best predictors for ventilation and death were the presence of air bronchogram (*p* = 0.006), crazy paving (*p* = 0.007), peripheral distribution (*p* < 0.001), age (*p* = 0.002), fever at admission (*p* = 0.007), dyspnea (*p* = 0.002) and cardiovascular comorbidities (*p* < 0.001). In multivariable analysis, quantitative CT parameters and features added incremental predictive value beyond a model with only clinical parameters (area under the curve, 0.78 vs. 0.74, *p* = 0.02). Our study demonstrates that quantitative evaluation of lung volume involved by COVID-19 pneumonia helps to predict patient’s clinical course.

## 1. Background

Lung infection named as COVID-19 pneumonia is an infectious disease caused by the most recently discovered severe acute respiratory syndrome coronavirus 2 (SARS-CoV-2) coronavirus. The outbreak that began in Wuhan, China, in December 2019, was declared a Public Health Emergency of International Concern on 30 January 2020 by the World health Organization (WHO). Imaging has shown, since the first cases described, a pivotal role in supporting the diagnosis of infection. Imaging and the RT-PCR test, are considered as the reference standard for final diagnosis [1]. In this scenario it has been shown that chest X-ray is burdened by low sensitivity in identifying the lung changes of COVID-19 in the early stages of the disease [2]. On the contrary chest CT (computed tomography) is considered as the first line imaging modality for suspected COVID-19, even in the initial stages given its high sensitivity [3,4]. COVID-19 pneumonia has various non-specific imaging features that can also be found in other lung infections, such as Influenza A (H1N1), Cytomegalovirus (CMV), other coronavirus (SARS, MERS), streptococcus and atypical pneumonias (such as Chlamydia or Mycoplasma). The most common findings in CT are bilateral multifocal “ground glass opacities” (GGO) associated with consolidations with patchy distribution, mainly peripheral/subpleural and with involvement of the lower lobes. Focal or multifocal GGO pattern alone and the “crazy paving” pattern were also observed. The presence of consolidations alone or of the “reversed halo sign” (focal area of GG delimited by peripheral ring with or without consolidation), cavitations, lymphadenopathies and pleural effusion have been less frequently described [5]. Moreover, CT has been demonstrated to have a better sensitivity in comparison with RT-PCR, particularly in early stages due to false negative results caused by sampling errors or low viral load [6]. However, CT findings appear to be highly variable and not always related to clinical severity and in particular to the degree of respiratory failure of the patient, with possible discrepancies between the extension of the lung infection and symptoms [7]. During the course of patient management, it is often necessary to repeat CT examinations in order to evaluate the progression or regression of COVID-19. Dedicated post processing software exists to allow a detailed evaluation of the lungs, discriminating between normal parenchyma and parenchyma affected by emphysema or other diseases by different Hounsfield Units (HU) thresholds [8,9]. Through appropriate variations of density threshold ranges it is possible to obtain differentiation between GGO or consolidations and that of healthy parenchyma areas. To the best of our knowledge no specific data is available, by literature, to discriminate between extension of the COVID-19 pneumonia and clinical symptoms. The aim of this study is to evaluate a correlation between the volume of lung parenchyma involved with COVID-19 infection (expressed as a percentage in comparison with the total lung volume) and the clinical outcome in a population of patients with cardiovascular disease. We aim to identify a specific lung involvement threshold that could predict the need for non-invasive or invasive ventilatory assistance or the onset of respiratory failure to improve the downstream management of these patients. Moreover, we analyzed subgroups with different comorbidities and blood tests to find further independent predictors for ventilatory assistance.

## 2. Methods

### 2.1. Ethical Standards

This retrospective study was approved by the local Ethical Board (IRB approval n. R1228/20-CCM 1295). Informed consent was obtained from all patients.

### 2.2. Patient Population

Patients from our emergency department or inpatients with suspected COVID-19 lung disease referred to the imaging department for Chest CT from 24 February to 6 April 2020 were included in the study. Patients without COVID-19 imaging features, with pneumonia other than COVID-19 or with contraindication to CT were excluded. Thus 76 patients were included in the study. Patients population characteristics are listed in Table 1. The overall mean age was 66.0 ± 14.4 years with 45 males and 31 females. In the overall population 47 patients out of 76 (62%) presented with dyspnea at admission and 42/76 patients (55%) presented with fever. Chest pain and muscle/joint pain were reported respectively in 15 and 25 patients out of 76. On admission, lymphocytopenia was present in 60% of the patients (46/76).

### 2.3. CT protocol, Images Reconstruction and Analysis

All CT examinations were performed using a 256-slice CT scanner (Revolution CT; GE Healthcare, Milwaukee, WI, USA) using the following parameters: helical caudo-cranial non gated acquisition with 100 kV peak tube voltage and mAs ranging from 250 to 450 depending on patients body mass index. Detector configuration, 256 × 0.625 mm; voxel size, 0.625 mm; gantry rotation time 280 msec and latest generation iterative reconstruction algorithm (ASIR-V; GE Healthcare) at 50%. No contrast media was administered. The mean scan time was 2.6 s. CT datasets of 76 patients with confirmed COVID 19 infection by RT-PCR test were processed by a dedicated workstation (ADW 4.6, GE Healthcare) using multi-planar reformats by two radiologist with >5 and 10 years of experience independently. CT’s were recorded as positive in the presence of viral pneumonia imaging features [5]. Specifically:

1. Ground glass opacities (GGO),
2. GGO distribution,
3. Consolidations,
4. Multilobe/subpleural involvement,
5. Lower lobes involvement,
6. Crazy paving
7. Air bronchogram,
8. Pleural effusion,
9. Lymph nodes with short axis > 10 mm.

Moreover, each CT dataset was further reconstructed using a specific segmentation software (Thoracic-VCAR software, GE Healthcare, Buc, France). This quantitative approach enables an automated assessment of the pulmonary infection depicting infection areas as high attenuation areas (HAAs) in respect of a defined threshold value ranging from −650 Hounsfield unit (HU) to 3071 HU. The specific threshold (−650 HU), was set to differentiate GGO and consolidated lung areas from the normal lung parenchyma, not affected by pathological findings. The amount of infected lung defined as the percentage of lung parenchyma above the predefined threshold of −650 (HAA%, HAA/total lung volume) was automatically calculated by the dedicated software for both lungs and the total infected lung volume was calculated for each patients as follow: Right lung HAA% + Left lung HAA%. All larger airways were at the same time excluded from the analysis. The density mask settings were as follow: blue for the range from −1024 to −650 HU and grey-black for the range from −650 to 3071 HU.

Images were visually revised and assessed for segmentation by two expert radiologists independently blinded to informations regarding patients clinical data, and corrections were performed if necessary [10]. For each patient at admission symptoms, cardiovascular risk factors, therapy and blood test such as leucocytes, hemoglobin, lymphocytopenia, platelets, glomerular filtration rate (e-GFR), B-type natriuretic peptide (BNP), polymerase chain reaction (PCR), procalcitonin test (PCT), Troponine (Tn) levels, and D-Dimer levels were assessed and compared with clinical course and outcome. Outcome measures were obtained using two combined end-points: ventilation + death.

### 2.4. Statistical Analysis

Statistical analysis was performed using MedCalc software 19.1. Continuous variables are expressed as mean ± SD, and discrete variables are expressed as absolute numbers and percentages. The difference between the groups were assessed by Mann-Whitney U test for continuous variables and Chi-square test or Fisher’s exact test for categorical variables. The outcome was defined by need for invasive ventilation and death. Categories from continuous variables were obtained using as threshold, the median value of the overall sample. For the additional CT categories, metrics were obtained using cutoffs derived from quartiles. Multivariable logistic regression analysis were used to test the association between potential predictors and the outcome. Factors for which *p* values were less than 0.1 in univariable analysis were used as candidate variables for multivariable approach. Clinical predictor factors and CT imaging features for which *p* values were less than 0.05 in univariable analysis were used to calculate a clinical score to be used as a variable for multivariate analysis. In particular, we dichotomized the continuous variables above/below the median and summed for each patient the single variables in order to have a score that could assume values from 0 (low risk) to 10 (high risk) (CLINICAL). To this, we added as variable for multivariate analysis the Total affected lung V% (VOLUME) and Systemic Inflammatory response Index as a surrogate of all laboratory test included in the manuscript (calculated by: platelet counts × neutrophil counts/lymphocyte counts) (INFLA) as suggested by recent publications to be a remarkable prognostic indicator to assess the in-hospital mortality and the development of respiratory complications in patients with COVID-19 [11,12]. The performance of the predictive models, singles and composite (MODEL 1 including all clinical, laboratory and CT variables) were assessed using receiver operating characteristic analysis (ROC), and the area under the curve (AUC) values were compared using the DeLong test. A *p* value < 0.05 was considered statistically significant.

## 3. Results

### 3.1. Demographic, Clinical and Laboratory Characteristics, Treatment

In the subgroup of patients that underwent ventilatory assistance + death (29 patients) patients were older with a mean age of 73.4 ± 10.8 years while in the subgroup of patients who died (7 patients) mean age was 84.4 ± 3.7 years.

In the subgroup of patients who underwent invasive ventilation or died lymphocytopenia was present in 72% and 86% respectively. Most of the patients had low levels of Hemoglobin (mean 12.6 ± 2 g/dL) and elevated levels of C-reactive protein (PCR) and BNP; less common were elevated levels of D-dimer (6% among all the patients) and of PCT; except for patients who died. This subgroup of patients showed average levels of PCT (0.57 ± 0.31 ng/mL) and also had lower levels of glomerular filtration rate (32.2 ± 19.4) in comparison with the overall population (67.6 ± 27.4).

In the overall population only 9 patients out of 76 (12%) had elevated Troponin levels. Patients who underwent invasive ventilation or who died had higher Troponin values in comparison with the overall population (24% and 28% respectively). Among the overall population, 53% had cardiovascular diseases (hypertension, dyslipidemia and diabetes were the most common). Table 1 also reports patients’ specific treatment during hospitalization. A majority of the patients (54%) received intravenous antibiotic therapy, and almost 40% received Hydroxychloroquine and anticoagulant therapy.

### 3.2. CT Features

CT findings are summarized in Table 1. Most of the patients in the overall population had typical COVID-19 CT features [5] of GGO and consolidations with peripheral distribution and multilobar involvement with vascular enlargement. All patients who underwent ventilatory assistance and or death showed multilobar involvement (100%) and a higher incidence of air bronchogram (*p* = 0.003). In the subgroup of patients who died a significant higher incidence of crazy paving pattern was observed in comparison with general population. In the overall population we found an average infected lung volume of 31.4 ± 26.3% while in the subgroup of patient who needed ventilator assistance and who died the volume of infected lung was significantly higher (*p* < 0.001) with average values of 41.4 ± 28.5% and 72.7 ± 36.2% respectively. A good agreement between the two readers was found for COVID-19 CT features assessment (k = 0.92). A good inter reader agreement was also found for lung volume analysis segmentation and corrections (k = 0.90).

### 3.3. Logistic Regression Analysis

Univariable logistic regression analysis results for clinical, laboratory and imaging parameters are reported in Table 2.

Best predictors for ventilation and death were age (*p* = 0.002), fever at admission (*p* = 0.007) and dyspnea (*p* = 0.002). Moreover, cardiovascular comorbidities were strongly correlated with death outcome (*p* < 0.001) and in particular diabetes and dyslipidemia. Other significant correlations were found with leucocytes levels (*p* = 0.006), kidney failure (*p* < 0.001), BNP levels (*p* = 0.047) and PCT (*p* = 0.024). No significant correlations were found among Tn levels, D-Dimer and outcome. Concerning CT features worse outcome was significantly correlated with the infected lung volume. Best predictors for ventilation and death were the presence of air bronchogram (*p* = 0.006), crazy paving (*p* = 0.007) and peripheral distribution (*p* < 0.001). Table 3 reports multivariable logistic regression analysis results regarding Odds Ratios for association of Clinical score, CT Parameters and Inflammatory index score with risk of ventilation and death. The model derived by clinical score (CLINICAL) showed good correlation with the primary composite endpoint such as the CT derived total lung volume involved by the infection (VOLUME). The systemic inflammatory index (INFLA) was instead mildly correlated with the composite primary endpoint: in the subgroup of patients that underwent ventilation and or death higher values were found (Table 1) even if without statistical significance.

**Figure 1:**
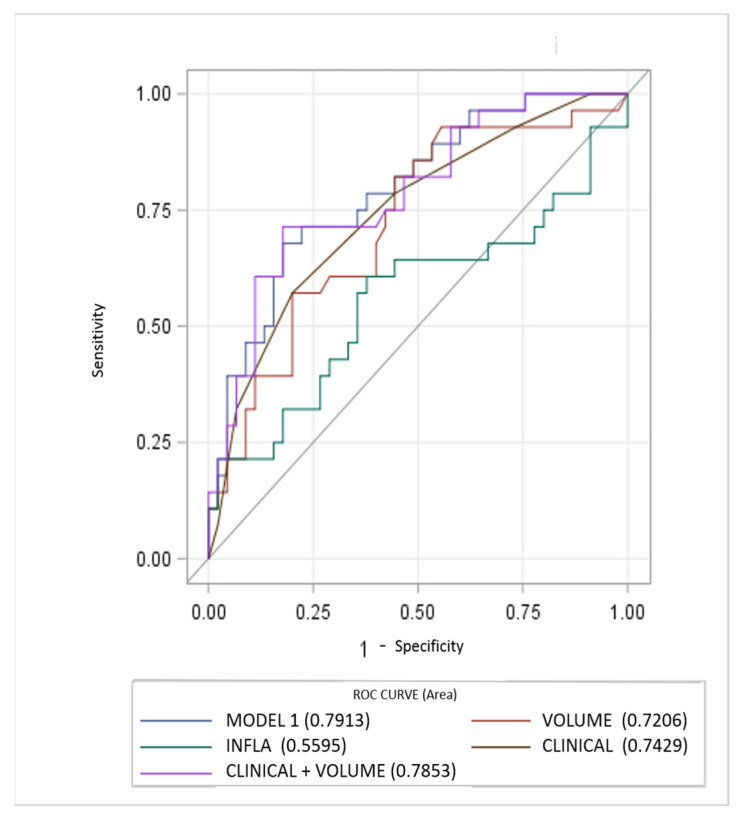
Performance of clinical, inflammatory and CT parameters (single and composite) for risk prediction of ventilator assistance and or death. CT measurements of lung involvement (VOLUME) add incremental predictive value to model including only clinical parameters (CLINICAL) and beyond a model only including laboratory features (INFLA).

Diagnostic performance of the models is reported in Figure 1. The MODEL 1 including all clinical, laboratory and imaging parameters resulted as most significant predictor at univariable analysis, showing higher AUC (0.7913, 95% CI, 0.68–0.89). AUC for the clinical score model (CLINICAL) was 0.7429 (95% CI, 0.62–0.85). Regarding the quantitative CT measurements (VOLUME) and Systemic Inflammatory response Index (INFLA) the AUC were 0.72 (95% CI, 0.59–0.84) and 0.55 (95% CI, 0.41–0.70) respectively. Incremental value in risk prediction was added by CT volume measurements above the CLINICAL model (AUC 0.7853, 95% CI, 0.67–0.89, *p* 0.02).

## 4. Discussion

**Figure 2:**
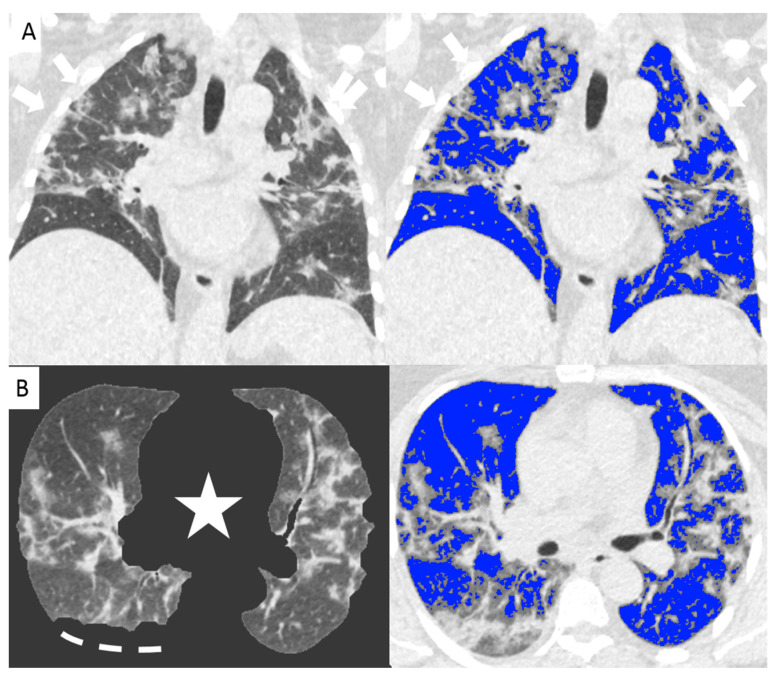
Non-enhanced coronal reconstructions of Chest CT (**A**), (upper panel) showing bilateral ground glass opacities with random distribution and consolidations in the upper and lower lobes (arrows). Lower panel show axial CT reconstructions of the same patient with automatic software segmentation (**B**) excluding all “non-lung” structures, (star) and not evaluable parenchyma due to pleural effusion (segmented line). Images on the right show software evaluation of the well aerated lung (blue) and the infected parenchyma (grey zone, arrows) discriminated by specific threshold. The whole infected volume assessed by software was 47% of the entire lung volume. The patient underwent invasive ventilation.

**Figure 3:**
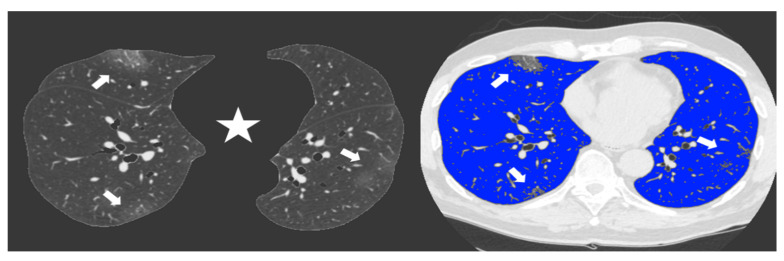
Non-enhanced axial Chest CT software reconstructions showing peripheral sub-pleural isolated GGOs (arrows). On the left the axial image automatically segmented by the software excluding all “non-lung” structures (mediastinum, upper airways and spine) (star) for visual quantification of infected parenchyma. On the right the same image highlighting in blue the well aerated lung and the COVID19 infection signs in grey. The whole infected volume assessed by software was 7% of the entire lung volume. The patient had good clinical course without need for invasive ventilation.

Since the beginning of the Covid-19 outbreak certain diagnosis of the disease was complicated by the multiplicity of symptoms and imaging features and due to the variability in the severity of disease at the time of presentation [1]. Chest CT has demonstrated an important role in predicting patients’ outcome because of the correlations between CT features and the severity of the disease [7,13]. In our study we demonstrated the correlation between the lung volume affected by the COVID-19 pneumonia and clinical outcome with a direct relationship between the infected lung percentage and the need for ventilation or subsequent death. Figure 2 and Figure 3 show lung volume analysis with dedicated software in comparison with standard CT images.

The main findings of the study can by summarized as follows: (a) The overall average value of infected lung volume in our population was 31.4 ± 26.3% while significantly higher volumes were observed in the subgroup of patient who underwent ventilator assistance and death (41.4 ± 28.5%) and who died prior to ventilator assistance (72.7 ± 36.2%) (*p* < 0.001); (b) A relevant proportion of patients (23/29, 80%) showed values >22% of the total lung volume; (c) Only one patient who needed ventilator assistance and died showed at admission absence of significant CT features with only two spotty isolated GGO < 1 cm. (d) Incremental value in risk prediction was added by CT volume measurements above laboratory or clinical models in multivariable analysis.

**Figure 4:**
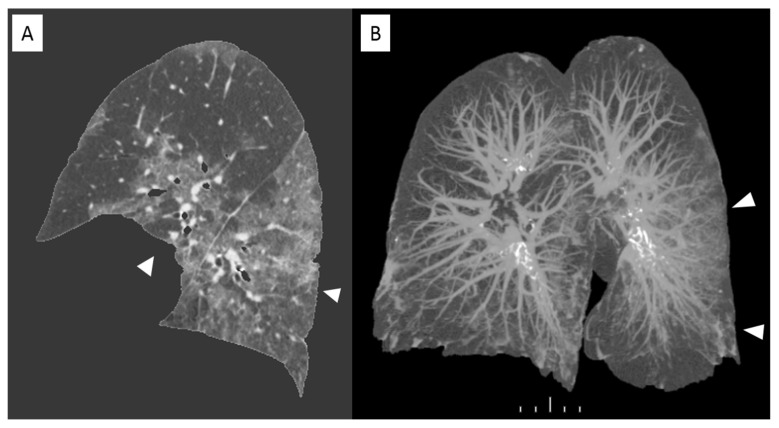
Sagittal view of a non-enhanced CT with software segmentation (**A**) showing extended GGOs (arrowheads). On the right the volume rendering recon of the same CT (**B**) highlighting the pulmonary vessels and the infected parenchyma hyperdense (arrowheads). in comparison with adjacent normal aerated lung.

Recent studies have demonstrated that in case of viral pneumonia inter-observer reliability for CT scans can be reduced for determining the presence of intra-lobular reticulation, distribution of consolidation, and GGO [14]. And this can be observed in case of COVID-19 assessment because of features overlapping. In this scenario computer-aided lung volume quantification has been demonstrated as a feasible way to stratify COVID-19 cases according to extension and severity [15]. Figure 4 shows a volume rendering reconstruction of the lung volume.

Our study results support these findings because despite good inter observer correlations for COVID-19 CT features assessment, the software aided evaluation of infected lung volume alone was directly correlated with patients outcome. Moreover, it is important to underline that we used a commercially and widely available software compatible with different CT scanners. Clinical conditions and laboratory findings are directly correlated with worst patient outcome and CT features should be adjusted with these parameters especially in our patients with high rate of cardiovascular diseases. Nevertheless, lung involvement alone seems to show a direct likelihood for ventilator assistance need and death. This can be related to previously demonstrated alveolar damage caused by virus invasion into pulmonary interstitium including alveolar edema and thickening of the interlobular interstitium [16] that can evolve to diffuse alveolar damage with cellular fibromyxoid exudate or due to perfusion defects as demonstrated in disease critical stages and that can result as GGO at chest CT [17,18]. Our results are consistent with these data and furthermore with the correlations found between specific CT features as predictors for ventilation and death such as air bronchogram and crazy paving pattern that have been demonstrated to be significantly higher in the severe or critical cases compared to the ordinary cases [19] and that may represent further infiltration signs of the lung parenchyma and interstitium [20]. Furthermore, our results are consistent with a recent study demonstrating a direct correlation between a software-based quantification of the well aerated lung on chest CT and the rate of intensive care unit admission or death in patients with COVID-19 pneumonia [21].

Some limitations of this study must be acknowledged. First this a single center retrospective study performed in a cardiovascular center with a smaller population in comparison with recently published studies. Second, we didn’t perform serial CT in all patients to assess lung changes in comparison with clinical course. Third some patients referred to our emergency department due to suspected COVID19 could have concomitant heart failure and this may have led to changes in lung interstitum mimicking more severe CT features.

In conclusion software-aided quantification of the lung volume involved by COVID-19 pneumonia on chest CT obtained at hospital admission associated with CT features are correlated with patients outcome and can help to stratify severe cases and predict the need for ventilatory assistance or subsequent death.

## Figures and Tables

**Table 1:** Patient population demographic, clinical and laboratory characteristics, treatment and CT findings.

	All Population (*n* = 76)	Ventilation + Death (*n* = 29)	*p*
**Clinical Features**			
Age years (mean ± sd)	66.0 ± 14.4	73.4 ± 10.8	<0.05
Sex (M/F)	45/31	19/10	0.2
BMI (kg/m^2^ (mean ± sd)	26.4 ± 4.3	27.1 ± 4.4	0.2
Fever at admission	42/76	11/29	0.06
Fatigue	20/76	20/29	0.3
dyspnea	47/76	25/29	0.5
Muscle joint pain	25/76	8/29	0.06
Chest pain	15/76	9/29	0.5
diarrhea	6/76	2/29	0.5
Hypertension	43/76	23/29	0.5
Dyslipidemia	31/76	17/29	0.5
Diabetes	20/76	13/29	0.5
Fam history	17/76	9/29	0.5
Smoke	11/76	6/29	0.5
CV disease	40/76	23/29	0.001
**Treatment during Hospitalization**			
Acetaminophen	19/76	12/29	n.a
Antibiotics	41/76	23/29	n.a
Plaquenil	31/76	9/29	n.a
antiviral	26/76	0/29	n.a
Steroids	8/76	3/29	n.a
anticoagulant	29/76	6/29	n.a
**Laboratory features**			
Leucocytes (10^3^/uL) (mean ± sd)	8.6 ± 4.8	10.7 ± 5.1	<0.05
Hemoglobin (g/dL) (mean ± sd)	12.6 ± 2	12.08 ± 2.2	<0.05
Linfocitopenia (y/n)	46/76	21/29	0.07
Platelets (10^3^/uL) (mean ± sd)	241.1 ± 118.5	264.8 ± 162.5	0.06
e-GFR (ml/min/1.73m^2^) (mean ± sd)	67.6 ± 27.4	52.5 ± 25.2	0.04
BNP (pg/mL) (mean ± sd)	507.7 ± 706.03	786.9 ± 910.7	<0.05
PCR (mg/L) (mean ± sd)	49.9 ± 57.9	63.1 ± 58.3	0.05
PCT (ng/mL) (mean ± sd)	0.23 ± 0.49	0.27 ± 0.29	<0.05
Increased Troponin (*n*)	9/76	7/29	0.6
Increased D-dimer (*n*)	5/76	4/29	0.7
**CT Features**			
Total infected lung V%	31.4 ± 26.3	41.4 ± 28.5	0.001
Normal lung V%	68.5 ± 26.4	41.8 ± 45.0	0.001
GGO + consolidation	34/76	17/29	0.004
Air bronchogram	20/76	13/29	0.003
Vascular enlargement	55/76	24/29	0.003
Crazy paving	25/76	15/29	0.08
Peripheral distribution	53/76	13/29	0.04
Multilobar involvement	67/76	29/29	0.8
SII	173.77 ± 93.17	196.48 ± 122.89	0.31

BMI: Body mass index; BNP: B-type natriuretic peptide; e-GFR: estimated glomerular filtration rate; GGO: ground glass opacities; PCR: polymerase chain reaction; PCT: procalcitonin test, SII: Systemic Inflammatory response Index; V: Volume; n.a.: not applicable.

**Table 2:** Logistic regression analysis for clinical, laboratory, therapeutic and CT features for predictors of ventilation and death.

	All Population (*n* = 76)	Ventilation + Death (*n* = 29)	*p*
Clinical features			
Age		1.06 (1.02–1.11)	0.002
Sex		1.53 (0.58–3.99)	0.381
BMI		1.01 (0.89–1.15)	0.786
Fever at admission		0.26 (0.09–0.69)	0.007
Fatigue		1.77 (0.49–6.34)	0.378
dyspnea		11.9 (2.51–56.71)	0.002
Muscle joint pain		-	
Chest pain		2.77 (0.86–8.91)	0.086
Diarrhea		-	
Hypertension		5.75 (1.85–17.83)	0.003
Dyslipidemia		3.42 (1.27–9.17)	0.014
Diabetes		4.71 (1.57–14.08)	0.006
Fam history		2.19 (0.72–6.59)	0.162
Smoke		1.85 (0.53–6.48)	0.331
CV disease		6.76 (2.30–19.87)	<0.001
Acetaminophen		-	-
Atb		6.76 (2.20–19.72)	<0.001
Plaquenil		0.55 (0.21–1.47)	0.239
antiviral		0.67 (0.24–1.85)	0.440
Steroids		4.68 (0.84–25.99)	0.077
anticoagulant		4.13 (1.53–11.09)	0.005
Laboratory features			
Leucocytes		1.16 (1.05–1.29)	0.006
Hemoglobin		0.80 (0.62–1.02)	0.079
Linfocitopenia (y/n)		2.31 (0.85–6.25)	0.099
Platelets		1.00 (0.99–1.01)	0.353
e-GFR		0.95 (0.93–0.98)	<0.001
BNP		1.01 (1.00–1.01)	0.047
PCR		1.01 (0.99–1.02)	0.052
PCT		51.42 (1.66–159.29)	0.024
Troponin		1.00 (0.99–1.019)	0.458
D-dimer		1.00 (0.99–1.01)	0.181
CT features			
Total lung vol %		1.03 (1.01–1.05)	0.006
GGO + consolidation		2.51 (0.96–6.459)	0.058
Bronchogram		4.64 (1.56–13.76)	0.006
Vascular enlargement		2.47 (0.79–7.72)	0.117
Crazy paving		3.96 (1.44–10.87)	0.007
Peripheral distribution		0.14 (0.04–0.42)	<0.001

BNP: B-type natriuretic peptide; e-GFR: estimated glomerular filtration rate; GGO: ground glass opacities; PCR: polymerase chain reaction; PCT: procalcitonin test; vol: Volume.

**Table 3:** Association of Clinical score, CT Parameters and Inflammatory index score with risk of ventilation and death in Multivariable Logistic Regression Analysis (Odds Ratios and Wald confidence intervals).

	Coefficient	OR (95% CI)	*p* Value
CLINICAL SCORE (CLINICAL)	1	1.75 (1.157–2.648)	0.008
INFLAMMATORY INDEX (INFLA)	1	1.003 (0.997–1.009)	0.397
Total lung volume (VOLUME)	1	1.025 (1.003–1.048)	0.026

CI: confidence interval; OR: Odds Ratio.

## Data Availability

The data presented in this study are available on request from the corresponding author.
